# Macronutrient Composition and Depressive Symptoms Among Working-Age Participants in a US National Health Survey: An Isocaloric Substitution Analysis

**DOI:** 10.7759/cureus.114949

**Published:** 2026-08-21

**Authors:** Naoki Choda

**Affiliations:** 1 Occupational Health, Itsuki Occupational Health Office, Kobe, JPN

**Keywords:** depressive symptoms, dietary protein, isocaloric substitution, macronutrient composition, nhanes, occupational health

## Abstract

Background: Depressive symptoms are common among working-age adults, and diet may be a modifiable correlate. Macronutrient composition has been linked to depressive symptoms, but usually one macronutrient at a time, and the few analyses of exchanges between them were confined to restricted populations. Protein, fat, and carbohydrate together account for almost all dietary energy, so a higher share of one implies a lower share of another. This study aimed to identify which exchange of energy between protein, fat, and carbohydrate, if any, is associated with depressive symptoms in working-age adults.

Methods: In a cross-sectional analysis of the National Health and Nutrition Examination Survey from August 2021 to August 2023, we analysed 2,125 participants aged 20-64 years with two complete 24-hour dietary recalls. Depressive symptoms were defined as a Patient Health Questionnaire-9 score of 10 or higher. Protein, fat, and carbohydrate were expressed as percentages of macronutrient-derived energy, summing to 100%. Logistic regression estimated odds ratios (ORs) across quartiles, and leave-one-out models estimated isocaloric substitution of 5% of macronutrient energy, adjusted for demographic, socioeconomic, and lifestyle factors and body mass index. Models were repeated by sex and employment status. Unweighted analyses were primary.

Results: Depressive symptoms were present in 277 participants (13.0%). The OR for the highest versus lowest quartile was 0.42 (95% CI: 0.28-0.63; P for trend < 0.001) for protein and 1.81 (1.24-2.65) for carbohydrate; fat showed no association. Replacing 5% of macronutrient energy from carbohydrate with protein was associated with lower odds (OR: 0.70, 0.59-0.84; survey-weighted: 0.76, 0.61-0.95), as was the same exchange from fat to protein (0.74, 0.60-0.90; survey-weighted: 0.78, 0.58-1.05), whereas exchanging carbohydrate for fat was not. Substitution estimates were similar by sex and among participants who were currently working (0.73, 0.58-0.92) and those who were not.

Conclusions: Among working-age adults, replacing carbohydrate or fat with protein was associated with lower odds of depressive symptoms. The design is cross-sectional, and prospective confirmation is needed.

## Introduction

Depression is among the leading causes of disability worldwide [1]. Among the factors that might be modified, diet is inexpensive to address and is already a target of workplace and primary care programmes directed at other health outcomes. Overall diet quality has been studied widely in relation to depression [2]. A systematic review of prospective studies reported that higher diet quality was associated with a lower risk of depression, although its authors judged the strength of that evidence to be very low, citing reverse causation and limitations of internal and construct validity [3].

Diet can also be described by its composition: the proportions of energy from protein, fat, and carbohydrate are clinically interpretable and potentially relevant to intervention. As nutrient intakes are correlated with total energy intake, these proportions should be distinguished from the amount eaten, which is the purpose of energy-adjusted modelling [4]. In an analysis of the 2005 to 2016 cycles of the National Health and Nutrition Examination Survey (NHANES), a higher protein share was associated with a lower prevalence of depression and a higher carbohydrate share with a higher prevalence; fat was not associated [5]. A meta-analysis of 13 cross-sectional studies likewise reported lower odds of depression at higher total protein intake, while its authors noted that the intake levels required to prevent depression remain to be established [6].

These analyses examined each macronutrient one at a time. Protein, fat, and carbohydrate together account for almost all dietary energy and therefore form compositional data: a higher share of one implies a lower share of another. A single-macronutrient estimate adjusted for total energy contrasts that macronutrient with the average of all other energy sources, and cannot indicate which of the others was displaced. The same protein coefficient is compatible with replacing carbohydrate, with replacing fat, or with replacing a mixture of the two. Isocaloric substitution models resolve this by entering all but one component together with total energy, so that each coefficient represents the exchange of a fixed amount of energy between two specified macronutrients [7].

These models have been applied widely in cardiometabolic epidemiology. In one example, in a prospective cohort, replacing 5% of energy from carbohydrate with plant protein or plant fat was associated with lower mortality, whereas replacing it with animal protein or animal fat showed positive or null associations [8]. As far as we could ascertain, however, this exchange has been examined in relation to depressive symptoms in only two studies, both in restricted populations. One studied 354 Iranian men [9] and the other 976 adults with type 1 diabetes [10]. A larger NHANES analysis applied this approach to depressive symptoms, but the exchange reported was between carbohydrate subtypes of differing quality [11].

The August 2021 to August 2023 cycle is the first complete NHANES cycle after field operations were suspended because of the COVID-19 pandemic, a period during which depressive symptoms in US adults have been reported to have increased [12]. Previous NHANES analyses of diet and depression have generally included all adults; one comprised 17,845 participants aged 18 years and older [13]. We restricted the analysis to adults aged 20-64 years, the range within which employment is most common and to which occupational mental-health strategies are most readily applied. We examined macronutrient composition and depressive symptoms in NHANES 2021-2023, using quartile and isocaloric substitution models to identify which exchange of energy, if any, is associated with depressive symptoms.

## Materials and methods

Study design and participants

We conducted a cross-sectional analysis of NHANES, which has surveyed the health, nutrition, and health-related behaviour of the US civilian non-institutionalised population since 1999 [14]. We used the August 2021 to August 2023 cycle. The survey protocol was approved by the National Center for Health Statistics (NCHS) Ethics Review Board (Protocol #2021-05); participants gave verbal consent before the household interview and written informed consent before the examination at the mobile examination centre. The present work is a secondary analysis of de-identified public-use data and required no further ethical approval. The study is reported in accordance with the Strengthening the Reporting of Observational Studies in Epidemiology (STROBE) guideline for cross-sectional studies [15].

**Figure 1:**
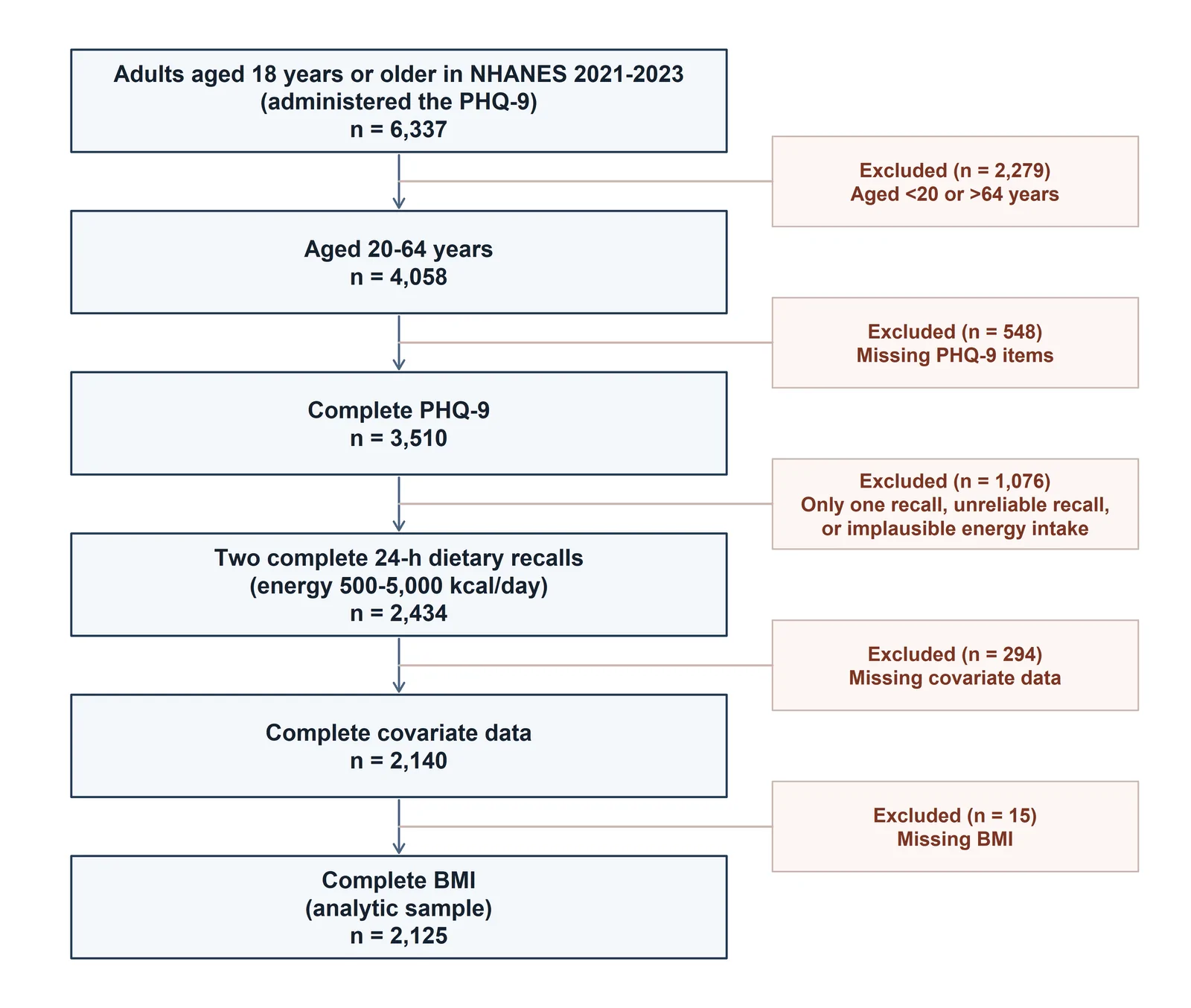
Sample selection flow chart. NHANES, National Health and Nutrition Examination Survey; PHQ-9, Patient Health Questionnaire-9; BMI, body mass index.

Of 6,337 participants who were administered the Patient Health Questionnaire-9 (PHQ-9) [16], we restricted the sample to 4,058 aged 20 to 64 years. We then excluded participants with incomplete PHQ-9 items (n = 548), leaving 3,510. We further required two complete and reliable 24-hour dietary recalls, a total energy intake between 500 and 5,000 kcal/day, and complete nutrient data (n = 1,076 excluded), then complete covariate data (n = 294) and a measured body mass index (n = 15). The analytic sample comprised 2,125 participants (Figure 1). No a priori sample size calculation was performed; the study size was determined by the number of participants in the cycle who met these criteria.

Depressive symptoms

Depressive symptoms were assessed with the PHQ-9, a nine-item instrument scored from 0 to 27. Consistent with common practice, a score of 10 or higher defined the presence of depressive symptoms; at this threshold, the instrument has a pooled sensitivity of 0.88 and a specificity of 0.85 against semi-structured diagnostic interviews [17]. Participants with any missing item were excluded rather than imputed.

Dietary assessment and macronutrient composition

Dietary intake was obtained from two 24-hour recalls collected with the US Department of Agriculture Automated Multiple-Pass Method [18]. Only recalls flagged by NCHS as reliable and meeting minimum criteria were used, and intakes were averaged across the two days. Energy from each macronutrient was calculated from the reported gram amounts using the general Atwater factors: protein and total carbohydrate at 4 kcal/g and total fat at 9 kcal/g [19]. Total carbohydrate as reported by NHANES includes dietary fibre and was used without subdivision. The exposure was the percentage of total macronutrient-derived energy contributed by each of the three, calculated by dividing the energy from that macronutrient by the sum of the energy from all three, so that the protein, fat, and carbohydrate shares sum to exactly 100% for every participant. Energy from alcohol was not included in this denominator. Alcohol was instead retained as a covariate, using intake reported on the alcohol use questionnaire rather than the dietary recalls. The denominator of the exposure was therefore the energy attributable to protein, fat, and carbohydrate, not total energy intake as reported by NHANES.

Covariates

Covariates were specified a priori, informed by the demographic, socioeconomic, and lifestyle factors used in previous analyses of diet and depressive symptoms [5,9,20]. Model 1 included age (continuous), sex, race/ethnicity (non-Hispanic White, Hispanic, non-Hispanic Black, non-Hispanic Asian, other or multiracial individuals) and total energy intake (continuous). Model 2 additionally included education (less than a bachelor's degree versus a bachelor's degree or above), current employment (yes or no), marital status (married or living with a partner, never married, separated, widowed or divorced), the family poverty income ratio, current smoking, alcohol consumption, sleep duration, leisure-time physical activity, and measured body mass index (continuous).

Alcohol consumption was categorised from the alcohol use questionnaire as none (fewer than 0.5 drinks per week), light (0.5 to fewer than 7), or moderate (7 or more). Average sleep duration was calculated from the reported weekday and weekend hours and categorised as <7, 7-9, or ≥9 hours. Leisure-time physical activity was categorised as <600, 600-1,199, or ≥1,200 metabolic equivalent of task (MET)-minutes per week, the lowest cut-point corresponding to the World Health Organization recommendation of 150 minutes of moderate-intensity activity per week [21]. Psychotropic medication, including antidepressants, could not be included as a covariate because the collection of individual prescription medication data was discontinued in this cycle [14].

Statistical analysis

All analyses were restricted to participants with complete data on the exposure, the outcome, and every covariate in Models 1 and 2, so no values were imputed and none of these variables had missing data within the analytic sample. Hours worked, which was used only in the stratified analysis of participants who were working, was the one exception and was available for 1,396 of them. Characteristics were compared between participants with and without depressive symptoms, and between the 2,125 included participants and the 1,933 age-eligible participants who were excluded, using Welch's t-test for continuous variables and the chi-square test for categorical variables.

Participants were divided into quartiles of each macronutrient share, and odds ratios with 95% confidence intervals were estimated by logistic regression, unadjusted and under Models 1 and 2, with the lowest quartile as the reference. Tests for trend were obtained by assigning each participant the median value of their quartile and entering this as a continuous term [20].

Isocaloric substitution was estimated with leave-one-out models [7]. Two of the three macronutrient shares were entered simultaneously with total energy intake and the covariates of Model 1 or Model 2, while the third was omitted; the coefficient for an entered macronutrient then represents the exchange of a fixed amount of energy from the omitted macronutrient for that macronutrient, at constant total energy. Unadjusted estimates, which include the two shares only, are reported alongside them. Coefficients were scaled to an exchange of 5% of macronutrient energy, which for a composition summing to 100% is equivalent to a change of five percentage points in the share of each macronutrient involved.

Stratified analyses

As the working-age focus of this study makes employment status of particular interest, the quartile and substitution models were repeated within four prespecified strata: men, women, participants who were currently working, and those who were not. Among those working, the models were additionally adjusted for the number of hours worked in the week before the interview.

Use of sampling weights

The primary analyses were unweighted, for the reasons set out in the Discussion. Survey weights are applied so that a survey sample can be used to produce nationally representative estimates; the survey weights and the design variables together account for the unequal probability of selection, for nonresponse, and for the clustered design [22]. In this cycle, oversampling was by age alone: all household members aged 0-19 years and 60 years and over were selected, and the number of adults aged 20-59 years selected depended on how many were resident in the household [22]. The whole-sample analyses were repeated with survey weights as a sensitivity analysis, using the two-day dietary weight with the survey strata and primary sampling units specified for this cycle [22].

Sensitivity analyses

Five sensitivity analyses were prespecified. First, the upper age limit was removed and all participants aged 20 years and older were included, to assess generalisability beyond the working-age range. Second, body mass index was removed from the model because obesity and depressive disorders are reciprocally associated in longitudinal studies [23], and body mass index may therefore lie on the causal path between diet and depressive symptoms. Third, the main analyses were repeated with survey weights. Fourth, the exposure was recalculated as percentages of the total energy intake reported in the survey, which includes energy from alcohol, in the conventional manner. Fifth, carbohydrate energy was recalculated counting dietary fibre at 2 kcal/g rather than 4 kcal/g, reflecting its incomplete digestion [19].

Software

Analyses were performed in R version 4.6.1 (R Foundation for Statistical Computing, Vienna, Austria), with survey-weighted models fitted using the survey package version 4.5 [24]. Two-sided P-values below 0.05 were considered statistically significant. The three macronutrients and the substitution contrasts were prespecified as the primary analyses; the stratified and sensitivity analyses were secondary.

## Results

Sample selection

Of 6,337 adults administered the PHQ-9, 2,125 met all inclusion criteria (Figure 1). Compared with the 1,933 age-eligible participants who were excluded, the 2,125 included participants were older (46.2 vs. 43.1 years, P < 0.001), more often non-Hispanic White (59.2% vs. 47.3%, P < 0.001), had a higher poverty income ratio (3.1 vs. 2.6, P < 0.001) and a lower prevalence of depressive symptoms (13.0% vs. 16.8%, P = 0.003). Body mass index (30.1 vs. 30.0 kg/m², P = 0.53) and total energy intake (2,028 vs. 2,082 kcal/day, P = 0.16) did not differ.

Participant characteristics

Depressive symptoms were present in 277 participants (13.0%). Compared with participants without depressive symptoms, those with depressive symptoms were younger and more often female, had less than a bachelor's degree, were more often not currently working, had a lower poverty income ratio, were more often current smokers, more often slept less than seven hours, more often reported low leisure-time physical activity, and had a higher body mass index (Table 1).

**Table 1:** Baseline characteristics of the study population. Values are mean (SD) for continuous variables and n (%) for categorical variables. P-values from Welch's t-test or the chi-square test; continuous comparisons were unchanged in rank-based tests. Macronutrient composition is the percentage of energy from protein, fat, and carbohydrate (Atwater factors 4, 9, and 4 kcal/g), which sums to 100% by definition.

Characteristic	Total (n = 2,125)	With depressive symptoms (n = 277)	Without depressive symptoms (n = 1,848)	P-value
Age, years, mean (SD)	46.2 (13.2)	43.7 (13.2)	46.5 (13.2)	0.001
Female, n (%)	1,219 (57.4)	183 (66.1)	1,036 (56.1)	0.002
Race/ethnicity, n (%)				
Non-Hispanic White	1,259 (59.2)	158 (57.0)	1,101 (59.6)	0.046
Hispanic	387 (18.2)	54 (19.5)	333 (18.0)	
Non-Hispanic Black	246 (11.6)	34 (12.3)	212 (11.5)	
Non-Hispanic Asian	118 (5.6)	8 (2.9)	110 (6.0)	
Other/Multiracial	115 (5.4)	23 (8.3)	92 (5.0)	
Less than a bachelor's degree, n (%)	1,231 (57.9)	200 (72.2)	1,031 (55.8)	<0.001
Not currently working, n (%)	627 (29.5)	124 (44.8)	503 (27.2)	<0.001
Marital status, n (%)				
Married/living with partner	1,142 (53.7)	95 (34.3)	1,047 (56.7)	<0.001
Never married	544 (25.6)	108 (39.0)	436 (23.6)	
Separated/widowed/divorced	439 (20.7)	74 (26.7)	365 (19.8)	
Poverty income ratio, mean (SD)	3.09 (1.70)	2.35 (1.62)	3.20 (1.68)	<0.001
Current smoker, n (%)	310 (14.6)	67 (24.2)	243 (13.1)	<0.001
Alcohol consumption, n (%)				
None (<0.5 drinks/week)	1,016 (47.8)	147 (53.1)	869 (47.0)	0.022
Light (0.5 to <7 drinks/week)	772 (36.3)	80 (28.9)	692 (37.4)	
Moderate (≥7 drinks/week)	337 (15.9)	50 (18.1)	287 (15.5)	
Sleep duration, n (%)				
Short (<7 h)	423 (19.9)	85 (30.7)	338 (18.3)	<0.001
Normal (7-9 h)	1,344 (63.2)	125 (45.1)	1,219 (66.0)	
Long (≥9 h)	358 (16.8)	67 (24.2)	291 (15.7)	
Leisure-time physical activity, n (%)				
Low (<600 MET-min/week)	951 (44.8)	163 (58.8)	788 (42.6)	<0.001
Moderate (600-1,199)	393 (18.5)	38 (13.7)	355 (19.2)	
High (≥1,200)	781 (36.8)	76 (27.4)	705 (38.1)	
Body mass index, kg/m², mean (SD)	30.1 (7.8)	31.4 (9.1)	30.0 (7.5)	0.012
Total energy intake, kcal/day, mean (SD)	2,028 (747)	1,904 (715)	2,047 (750)	0.002
Macronutrient composition, % of macronutrient energy, mean (SD)				
Protein	15.8 (4.5)	14.5 (4.6)	16.0 (4.4)	<0.001
Fat	38.5 (7.8)	37.2 (8.6)	38.7 (7.7)	0.006
Carbohydrate	45.7 (9.5)	48.3 (10.7)	45.3 (9.2)	<0.001

Total energy intake was lower among participants with depressive symptoms (1,904 vs. 2,047 kcal/day, P = 0.002). The macronutrient composition also differed: participants with depressive symptoms obtained a smaller share of macronutrient energy from protein (14.5% vs. 16.0%, P < 0.001) and from fat (37.2% vs. 38.7%, P = 0.006), and a larger share from carbohydrate (48.3% vs. 45.3%, P < 0.001).

Macronutrient composition by quartile

The prevalence of depressive symptoms fell across quartiles of the protein share, from 18.8% in the lowest quartile to 8.3% in the highest, and rose across quartiles of the carbohydrate share, from 10.2% to 19.4% (Table 2). In Model 2, the odds ratio for the highest versus the lowest quartile was 0.42 (95% CI: 0.28-0.63) for protein and 1.81 (1.24-2.65) for carbohydrate, with P for trend < 0.001 and 0.004, respectively. Estimates for protein and carbohydrate were similar in the crude model and in Model 1. Fat behaved differently: the highest quartile was associated with lower odds in the crude model (0.69, 0.49-0.98; P for trend = 0.027) and in Model 1 (0.68, 0.47-0.96; P for trend = 0.021), but the association was attenuated to the null after full adjustment (0.84, 0.58-1.22; P for trend = 0.302).

**Table 2:** Odds ratios for depressive symptoms by quartile of macronutrient composition. Model 1: age, sex, race/ethnicity, and total energy intake. Model 2: Model 1 plus education, employment, marital status, poverty income ratio, smoking, alcohol consumption, sleep duration, physical activity, and body mass index. P for trend was obtained by assigning the median value of each quartile and modelling it as a continuous variable.

Macronutrient and model	Quartile 1	Quartile 2	Quartile 3	Quartile 4	P for trend
Protein, % of macronutrient energy					
Range, %E	2.9-12.8	12.8-15.3	15.3-18.1	18.1-39.8	
Median, %E	11.3	14.0	16.6	20.3	
Cases/participants	100/532	69/531	64/531	44/531	
Crude	1.00 (Ref)	0.65 (0.46-0.90)	0.59 (0.42-0.83)	0.39 (0.27-0.57)	<0.001
Model 1	1.00 (Ref)	0.65 (0.47-0.92)	0.60 (0.43-0.85)	0.36 (0.24-0.53)	<0.001
Model 2	1.00 (Ref)	0.68 (0.48-0.97)	0.70 (0.49-1.01)	0.42 (0.28-0.63)	<0.001
Fat, % of macronutrient energy					
Range, %E	10.4-33.6	33.6-38.7	38.7-43.5	43.5-67.0	
Median, %E	29.5	36.2	41.0	47.0	
Cases/participants	89/532	63/531	60/531	65/531	
Crude	1.00 (Ref)	0.67 (0.47-0.95)	0.63 (0.45-0.90)	0.69 (0.49-0.98)	0.027
Model 1	1.00 (Ref)	0.64 (0.45-0.91)	0.61 (0.42-0.87)	0.68 (0.47-0.96)	0.021
Model 2	1.00 (Ref)	0.75 (0.52-1.09)	0.74 (0.51-1.09)	0.84 (0.58-1.22)	0.302
Carbohydrate, % of macronutrient energy					
Range, %E	1.5-39.8	39.8-45.6	45.6-51.8	51.8-82.8	
Median, %E	35.5	43.0	48.4	56.1	
Cases/participants	54/532	62/531	58/531	103/531	
Crude	1.00 (Ref)	1.17 (0.80-1.72)	1.09 (0.73-1.61)	2.13 (1.49-3.04)	<0.001
Model 1	1.00 (Ref)	1.19 (0.81-1.76)	1.08 (0.72-1.60)	2.19 (1.53-3.14)	<0.001
Model 2	1.00 (Ref)	1.27 (0.84-1.90)	1.01 (0.67-1.53)	1.81 (1.24-2.65)	0.004

Isocaloric substitution

In Model 2, replacing 5% of macronutrient energy from carbohydrate with protein was associated with lower odds of depressive symptoms (odds ratio: 0.70, 95% CI: 0.59-0.84; P < 0.001). The same exchange from fat to protein was associated with a comparable reduction (0.74, 0.60-0.90; P = 0.004). In contrast, exchanging the same amount of energy between carbohydrate and fat was not associated with depressive symptoms (0.96, 0.88-1.04; P = 0.321). The estimates for the two exchanges involving protein were similar before adjustment and under Model 1 (Table 3).

**Table 3:** Isocaloric substitution models for depressive symptoms. Protein, fat, and carbohydrate energy shares sum to 100% by definition, so the three contrasts are internally consistent. Models are as in Table 2: the crude model includes the two macronutrient shares only, and total energy intake is included from Model 1 onwards.

Isocaloric substitution	Crude odds ratio (95% CI)	P-value	Model 1 odds ratio (95% CI)	P-value	Model 2 odds ratio (95% CI)	P-value
Carbohydrate to protein, 5% of macronutrient energy	0.67 (0.56-0.79)	<0.001	0.66 (0.55-0.78)	<0.001	0.70 (0.59-0.84)	<0.001
Carbohydrate to fat, 5% of macronutrient energy	0.91 (0.84-0.99)	0.025	0.92 (0.84-1.00)	0.045	0.96 (0.88-1.04)	0.321
Fat to protein, 5% of macronutrient energy	0.73 (0.60-0.89)	0.002	0.71 (0.58-0.88)	0.001	0.74 (0.60-0.90)	0.004

Stratified analyses

Under Model 2, the carbohydrate-to-protein estimate was similar in all four strata: 0.70 (0.53-0.94) in 906 men and 0.70 (0.56-0.87) in 1,219 women, and 0.73 (0.58-0.92) in the 1,498 participants who were currently working and 0.67 (0.51-0.89) in the 627 who were not. Among those working, the estimate was unchanged when hours worked in the previous week were added to Model 2, which were recorded for 1,396 of them (0.72, 0.57-0.91). The quartile estimates for the protein share were also similar: 0.43 (0.25-0.75) among those working and 0.32 (0.17-0.61) among those who were not. Those for the carbohydrate share were less consistent, reaching conventional significance in men (2.62, 1.34-5.14) and in those not working (2.43, 1.31-4.52) but not in women (1.55, 0.97-2.49) or those working (1.52, 0.93-2.47) (Table 4).

**Table 4:** Isocaloric substitution and quartile estimates by sex and employment status. All estimates are unweighted and adjusted as in Model 2, omitting the stratifying variable. For participants who were currently working, hours worked in the previous week were added to Model 2. Substitution estimates are per 5% of macronutrient energy. Quartiles were defined within each stratum.

Stratum	Participants	Cases	Carbohydrate to protein odds ratio (95% CI)	Fat to protein odds ratio (95% CI)	Carbohydrate to fat odds ratio (95% CI)	Protein, highest vs. lowest quartile odds ratio (95% CI) (P for trend)	Carbohydrate, highest vs. lowest quartile odds ratio (95% CI) (P for trend)
Men	906	94	0.70 (0.53-0.94)	0.79 (0.56-1.12)	0.89 (0.77-1.03)	0.41 (0.21-0.81) (0.005)	2.62 (1.34-5.14) (0.004)
Women	1,219	183	0.70 (0.56-0.87)	0.69 (0.53-0.90)	1.00 (0.90-1.13)	0.43 (0.26-0.71) (0.004)	1.55 (0.97-2.49) (0.121)
Currently working	1,498	153	0.73 (0.58-0.92)	0.75 (0.57-0.98)	0.98 (0.87-1.10)	0.43 (0.25-0.75) (0.004)	1.52 (0.93-2.47) (0.075)
Currently working, adjusted for working hours	1,396	146	0.72 (0.57-0.91)	0.74 (0.56-0.99)	0.97 (0.86-1.10)	0.39 (0.22-0.70) (0.002)	1.55 (0.94-2.55) (0.068)
Not currently working	627	124	0.67 (0.51-0.89)	0.73 (0.53-1.01)	0.92 (0.80-1.06)	0.32 (0.17-0.61) (0.002)	2.43 (1.31-4.52) (0.011)

Sensitivity analyses

The carbohydrate-to-protein substitution was stable across the sensitivity analyses. It was 0.71 (0.59-0.84) when body mass index was removed from the model, and 0.71 (0.59-0.84) when alcohol was included in the energy denominator so that the shares were expressed as percentages of the total energy reported in the survey. It was 0.71 (0.60-0.84) when dietary fibre was assigned 2 kcal/g rather than 4 kcal/g in the calculation of carbohydrate energy. The quartile estimates were likewise unchanged when body mass index was removed (highest versus lowest quartile of protein: 0.42, 0.28-0.63; carbohydrate: 1.79, 1.23-2.62). Extending the sample to all adults aged 20 years and older (n = 3,324, of whom 361 (10.9%) had depressive symptoms) attenuated the estimates but preserved their direction: the carbohydrate-to-protein substitution was 0.73 (0.63-0.85), the odds ratio for the highest versus the lowest quartile of protein was 0.49 (0.35-0.69) and that for carbohydrate was 1.49 (1.07-2.06).

In survey-weighted Model 2 analyses, the carbohydrate-to-protein estimate was preserved (0.76, 0.61-0.95; P = 0.018) and the fat-to-protein estimate was similar in size but no longer significant (0.78, 0.58-1.05; P = 0.100). The quartile estimate for protein was attenuated and no longer statistically significant (highest versus lowest quartile: 0.57, 0.27-1.21; P for trend = 0.119), whereas that for carbohydrate remained significant (1.61, 1.20-2.16; P for trend = 0.036).

## Discussion

In this cross-sectional analysis of working-age NHANES 2021-2023 participants, macronutrient composition was associated with depressive symptoms, and the association was not symmetric across the three macronutrients. A higher share of energy from protein and a lower share from carbohydrate were each associated with lower odds of depressive symptoms, whereas the fat share showed no association after full adjustment. The substitution models made the structure of this pattern explicit: replacing carbohydrate with protein, and replacing fat with protein, were both associated with lower odds, while exchanging energy between carbohydrate and fat was not. Taken together, these results locate the association in exchanges involving protein rather than in the balance between carbohydrate and fat.

The direction of our findings agrees with a previous analysis of 15,296 NHANES participants, which reported an inverse association for protein, a positive association for carbohydrate, and no association for fat [5]. That analysis entered only one macronutrient at a time because the three shares are collinear, so its estimates could not specify which macronutrient was displaced. We instead used the leave-one-out formulation, in which one share is omitted so that the other two can be entered together and their coefficients read as substitutions [7]. In the two models fitted, the shares entered together were only weakly correlated, with variance inflation factors of 1.7 or below.

The direction of our findings also agrees with a meta-analysis of cross-sectional studies, which reported an odds ratio of 0.56 comparing the highest with the lowest total protein intake and a 21% lower risk per 10% increment in energy from protein [6]. A separate NHANES analysis of 23,204 adults approached the question differently, summarising the three shares in a single low-carbohydrate-diet score; a higher score and a higher protein share were both associated with lower odds of depression [25].

As far as we could ascertain, the exchange between the three macronutrients has been examined in relation to mental health in only two previous studies, both in restricted populations. In 354 Iranian men, substituting carbohydrate with protein was associated with better sleep quality; for depressive symptoms, the corresponding estimate was in the same direction but not significant (0.90, 95% CI: 0.67-1.20, per 3% of energy) [9]. That study was smaller than ours and used a different depression scale. In 976 adults with type 1 diabetes, favouring protein over carbohydrate or fat was associated with lower depression scores [10]; the direction is the same as ours.

Mechanisms have been proposed for protein and for carbohydrate separately, although the present design cannot test them. Tryptophan, phenylalanine, and tyrosine are dietary precursors of the monoamines serotonin, dopamine and noradrenaline, which is one reason why protein intake may be relevant to depression [2]. For carbohydrate, the proposed pathway is glycaemic: the compensatory hyperinsulinaemia that follows a high glycaemic load may lower plasma glucose enough to trigger counter-regulatory hormones such as adrenaline and cortisol, which can alter mood [26].

Weighted and unweighted estimation

As our objective was to estimate the association within the analytic sample rather than a nationally representative population parameter, the primary analyses were unweighted, and weighted analyses were performed as a sensitivity analysis. Weighted regression is the standard approach for complex survey data, although it has limitations and alternatives [27]. Weights that are applied unnecessarily can produce an inefficient estimator without reducing bias [28], and precision may fall further in subgroups, for which NHANES itself advises that standard errors are larger and confidence intervals wider [22]. Age, the variable used for oversampling, is adjusted for in every model. Selection within a household also depended on the number of adults aged 20-59 living there [14], and adding household size to Model 2 changed the substitution estimates by no more than 2%. The carbohydrate-to-protein substitution was preserved when weights were applied.

Implications for occupational health

The association was of similar size among men and women, and among those working and those not working, and among workers it persisted after adjustment for weekly working hours. Two features make this relevant to occupational health practice. First, dietary programmes are already delivered in workplaces to reduce risk factors for noncommunicable disease [29], and macronutrient composition could be addressed alongside them through canteens, catering contracts, and health guidance. Second, working conditions may themselves shape the macronutrient shares: in a Korean survey, shift workers reported eating protein-rich foods less often than day workers [30], so shift schedules may indicate who is most likely to have a low protein share. Our estimates cannot establish that changing composition changes symptoms, but they indicate where such an effort would be directed.

Strengths and limitations

The main strength of this study is the isocaloric substitution analysis. As the protein, fat, and carbohydrate shares sum to 100% of macronutrient energy, it indicates which exchange the association is located in rather than leaving the displaced macronutrient unspecified. The analysis was also confined to the working-age range, in which occupational health practice operates, and reported estimates by sex and employment status. It used the first complete NHANES cycle collected since the pandemic began, required two 24-hour dietary recalls rather than one, and was examined in five prespecified sensitivity analyses, one of which applied the survey weights.

This study also has several limitations. First, its cross-sectional design precludes causal inference, and reverse causation remains possible because reduced appetite is a symptom of depression. In a cohort analysed both ways, the association between dietary protein and poor mental health was weaker prospectively [20], so prospective studies are needed. Second, dietary intake was assessed from two 24-hour recalls and remains susceptible to measurement error. Third, nearly half of the age-eligible participants were excluded, most often because two reliable recalls were required. Completing them may be related to both diet and depressive symptoms, so selection may have biased the association as well as limiting generalisability. Fourth, the mean body mass index in the analytic sample was 30.1 kg/m², and the findings may not extend to leaner working populations. Fifth, we did not distinguish protein sources, fatty acid composition, or carbohydrate quality, and residual confounding cannot be excluded (including antidepressant use, which cannot be identified in this cycle). Nevertheless, the substitution estimates were consistent across the five sensitivity analyses, suggesting that they are not an artefact of the analytical choices made.

## Conclusions

Among working-age adults participating in NHANES 2021-2023, a higher share of dietary energy from protein was associated with lower odds of depressive symptoms. Substituting protein for either carbohydrate or fat was associated with lower odds, whereas exchanging carbohydrate for fat was not. These are cross-sectional associations, and prospective studies are required before any dietary recommendation can be drawn from them.

## References

[1] GBD 2019 Mental Disorders Collaborators (2022). Global, regional, and national burden of 12 mental disorders in 204 countries and territories, 1990-2019: a systematic analysis for the Global Burden of Disease Study 2019. Lancet Psychiatry.

[2] Kunugi H (2023). Depression and lifestyle: focusing on nutrition, exercise, and their possible relevance to molecular mechanisms. Psychiatry Clin Neurosci.

[3] Molero P, De Lorenzi F, Gędek A (2025). Diet quality and depression risk: a systematic review and meta-analysis of prospective studies. J Affect Disord.

[4] Willett WC, Howe GR, Kushi LH (1997). Adjustment for total energy intake in epidemiologic studies. Am J Clin Nutr.

[5] Oh J, Yun K, Chae JH, Kim TS (2020). Association between macronutrients intake and depression in the United States and South Korea. Front Psychiatry.

[6] Fotouhi Ardakani A, Shirdeli M, Khorvash F, Hajhashemy Z, Askari G (2026). Association between total, animal, and plant protein intake and risk of depression in adults: a systematic review and dose-response meta-analysis of observational studies. Nutr Rev.

[7] Tomova GD, Gilthorpe MS, Tennant PW (2022). Theory and performance of substitution models for estimating relative causal effects in nutritional epidemiology. Am J Clin Nutr.

[8] Zeng X, Li X, Zhang Z (2022). A prospective study of carbohydrate intake and risk of all-cause and specific-cause mortality. Eur J Nutr.

[9] Mohammadi S, Ghasemi M, Parastouei K, Hosseini SM, Eskandari E (2025). Association between isocaloric substitution of macronutrient intake with sleep disorders and psychological health in male adults. J Res Med Sci.

[10] Ahola AJ, Forsblom C, Groop PH (2018). Association between depressive symptoms and dietary intake in patients with type 1 diabetes. Diabetes Res Clin Pract.

[11] Song X, He K, Xu T (2024). Association of macronutrient consumption quality, food source and timing with depression among US adults: a cross-sectional study. J Affect Disord.

[12] Ettman CK, Cohen GH, Abdalla SM (2022). Persistent depressive symptoms during COVID-19: a national, population-representative, longitudinal study of U.S. adults. Lancet Reg Health Am.

[13] Li Y, Zhang C, Li S, Zhang D (2020). Association between dietary protein intake and the risk of depressive symptoms in adults. Br J Nutr.

[14] Terry AL, Chiappa MM, McAllister J, Woodwell DA, Graber JE (2024). Plan and operations of the National Health and Nutrition Examination Survey, August 2021-August 2023. Vital Health Stat 1.

[15] von Elm E, Altman DG, Egger M, Pocock SJ, Gøtzsche PC, Vandenbroucke JP (2007). The Strengthening the Reporting of Observational Studies in Epidemiology (STROBE) statement: guidelines for reporting observational studies. Ann Intern Med.

[16] Kroenke K, Spitzer RL, Williams JB (2001). The PHQ-9: validity of a brief depression severity measure. J Gen Intern Med.

[17] Levis B, Benedetti A, Thombs BD (2019). Accuracy of Patient Health Questionnaire-9 (PHQ-9) for screening to detect major depression: individual participant data meta-analysis. BMJ.

[18] Moshfegh AJ, Rhodes DG, Baer DJ (2008). The US Department of Agriculture Automated Multiple-Pass Method reduces bias in the collection of energy intakes. Am J Clin Nutr.

[19] (2026). Food and Agriculture Organization of the United Nations. Food energy - methods of analysis and conversion factors. FAO Food and Nutrition Paper.

[20] Choda N, Wakai K, Naito M (2020). Associations between diet and mental health using the 12-item General Health Questionnaire: cross-sectional and prospective analyses from the Japan Multi-Institutional Collaborative Cohort Study. Nutr J.

[21] Bull FC, Al-Ansari SS, Biddle S (2020). World Health Organization 2020 guidelines on physical activity and sedentary behaviour. Br J Sports Med.

[22] (2026). National Center for Health Statistics. Brief overview of sample design, nonresponse bias assessment, and analytic guidelines for NHANES August 2021-August 2023. https://wwwn.cdc.gov/nchs/nhanes/continuousnhanes/overviewbrief.aspx?Cycle=2021-2023.

[23] Luppino FS, de Wit LM, Bouvy PF, Stijnen T, Cuijpers P, Penninx BW, Zitman FG (2010). Overweight, obesity, and depression: a systematic review and meta-analysis of longitudinal studies. Arch Gen Psychiatry.

[24] Lumley T (2004). Analysis of complex survey samples. J Stat Softw.

[25] Cheng Z, Fu F, Lian Y, Zhan Z, Zhang W (2024). Low-carbohydrate-diet score, dietary macronutrient intake, and depression among adults in the United States. J Affect Disord.

[26] Gangwisch JE, Hale L, Garcia L (2015). High glycemic index diet as a risk factor for depression: analyses from the Women's Health Initiative. Am J Clin Nutr.

[27] Lumley T, Scott A (2017). Fitting regression models to survey data. Stat Sci.

[28] Bollen KA, Biemer PP, Karr AF, Tueller S, Berzofsky ME (2016). Are survey weights needed? A review of diagnostic tests in regression analysis. Annu Rev Stat Appl.

[29] Turon H, Bezzina A, Lamont H (2024). Interventions in the workplace to reduce risk factors for noncommunicable diseases: an umbrella review of systematic reviews of effectiveness. J Occup Health.

[30] Kim CH, Lee W (2025). Shift work and dietary behaviors among Korean workers. J Occup Health.

